# Supplementary material for: Epidermal growth factor receptor signaling uncouples germ cells from the somatic follicular compartment at ovulation
Source: Nat Commun. 2021 Mar 4;12:1438. doi: 10.1038/s41467-021-21644-z (PMC7933413; doi:10.1038/s41467-021-21644-z)
Supplement: Supplementary file 1 — Supplementary Information [file 41467_2021_21644_MOESM1_ESM.pdf]

Supplementary Figure 1

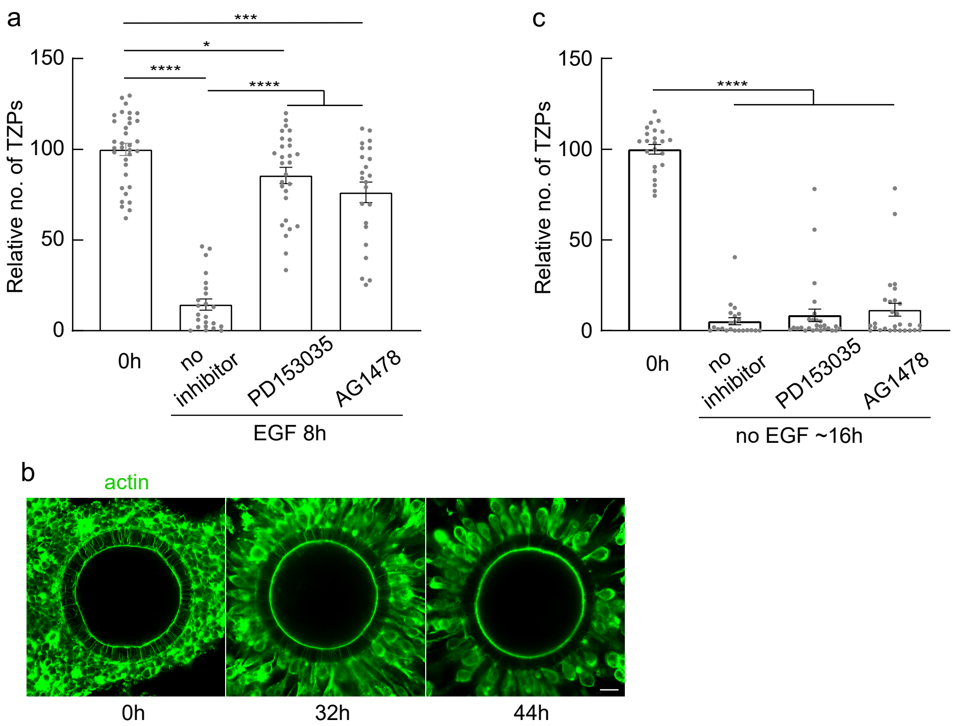

Supplementary Figure 1. Loss of TZPs during maturation of cumulus-oocyte complexes. a Mean and SEM of number of TZPs, normalized to the number at 0 h, in COCs exposed to 10 ng/ml EGF for 8 h in the presence or absence of the indicated EGFR inhibitors. Each point represents an individual COC. n = 34 (0 h), 23 (8 h EGF), 28 (8 h EGF PD153035), 23 (8 h EGF AG1478) COCs examined over three independent experiments. Statistical analysis using one-way ANOVA with Tukey multiple comparison test. **** P<0.0001 *** P=0.0005 * P=0.0497. **b** Confocal images of phalloidin-stained porcine COCs at indicated times during maturation in vitro. TZPs are lost even though cumulus cells remain adjacent to the oocyte. Scale bar = 20 µm. **c** Mean and SEM of number of TZPs, normalized to the number at 0 h, in COCs incubated in EGF-free medium for ~16 h in the presence or absence of the indicated inhibitors. Each point represents an individual COC. n = 23 (0 h), 22 (16 h), 27 (16 h PD153035), 28 (16 h AG1478) COCs examined over three independent experiments. Statistical analysis using one-way ANOVA with Tukey multiple comparison test. **** P<0.0001.

Supplementary Figure 2

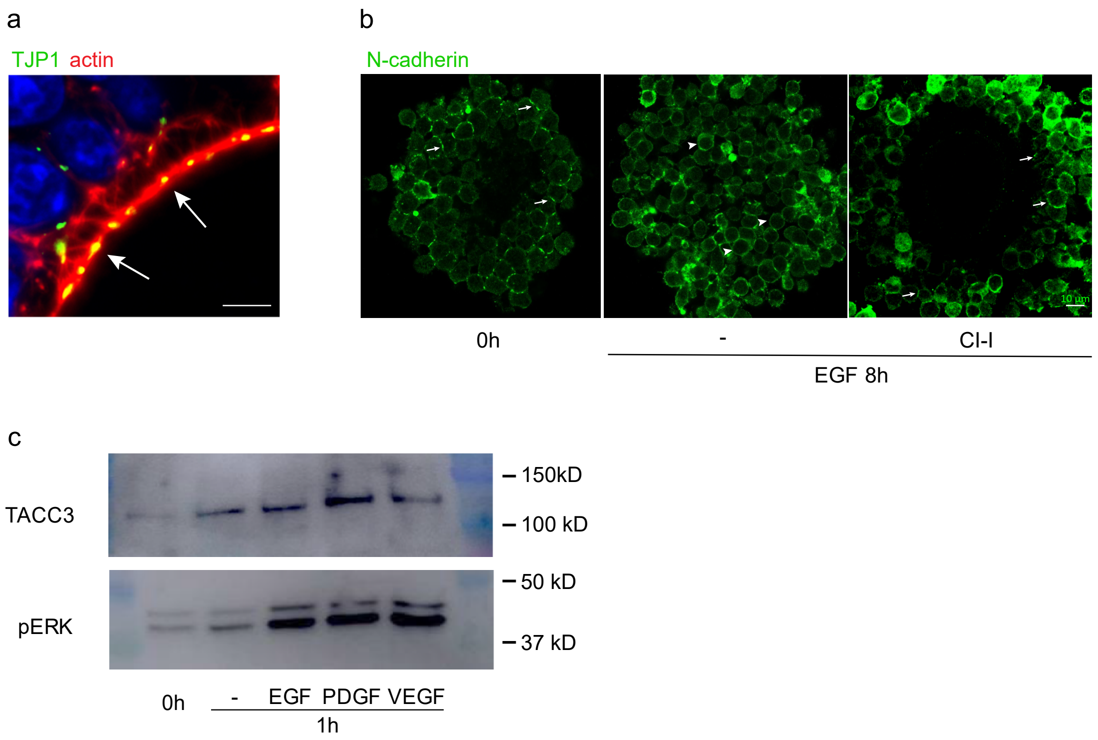

Supplementary Figure 2.Mechanisms regulating TZP retraction. a Portion of a granulosa-oocyte complex stained using anti-TJP1 (green) and phalloidin (red). **b** Confocal image of cumulus cells within COCs under the indicated conditions. Arrows show N-cadherin foci in some cells of the 0h and EGF + CI-I groups. Arrowhead shows cytoplasmic staining in the EGF group. **c** Immunoblot of granulosa cells obtained from granulosa cell-oocyte complexes (GOCs) incubated for 1h in the presence of the indicated growth factors. Scale bar = 5 μm (a), 10 μm (b).
