# Supplementary material for: Epidermal growth factor receptor signaling uncouples germ cells from the somatic follicular compartment at ovulation
Source: Nat Commun. 2021 Mar 4;12:1438. doi: 10.1038/s41467-021-21644-z (PMC7933413; doi:10.1038/s41467-021-21644-z)
Supplement: Supplementary file 3 — Reporting Summary [file 41467_2021_21644_MOESM3_ESM.pdf]

nature research

Corresponding author(s): Hugh Clarke

Last updated by author(s): Jan 14, 2021

# Reporting Summary

Nature Research wishes to improve the reproducibility of the work that we publish. This form provides structure for consistency and transparency in reporting. For further information on Nature Research policies, see our Editorial Policies and the Editorial Policy Checklist.

## Statistics

For all statistical analyses, confirm that the following items are present in the figure legend, table legend, main text, or Methods section.

| n/a | Confirmed | |
|---|---|---|
| ☐ | ☒ | The exact sample size (*n*) for each experimental group/condition, given as a discrete number and unit of measurement |
| ☐ | ☒ | A statement on whether measurements were taken from distinct samples or whether the same sample was measured repeatedly |
| ☐ | ☒ | The statistical test(s) used AND whether they are one- or two-sided<br>*Only common tests should be described solely by name; describe more complex techniques in the Methods section.* |
| ☐ | ☒ | A description of all covariates tested |
| ☐ | ☒ | A description of any assumptions or corrections, such as tests of normality and adjustment for multiple comparisons |
| ☐ | ☒ | A full description of the statistical parameters including central tendency (e.g. means) or other basic estimates (e.g. regression coefficient) AND variation (e.g. standard deviation) or associated estimates of uncertainty (e.g. confidence intervals) |
| ☐ | ☒ | For null hypothesis testing, the test statistic (e.g. *F*, *t*, *r*) with confidence intervals, effect sizes, degrees of freedom and *P* value noted<br>*Give P values as exact values whenever suitable.* |
| ☒ | ☐ | For Bayesian analysis, information on the choice of priors and Markov chain Monte Carlo settings |
| ☒ | ☐ | For hierarchical and complex designs, identification of the appropriate level for tests and full reporting of outcomes |
| ☒ | ☐ | Estimates of effect sizes (e.g. Cohen's *d*, Pearson's *r*), indicating how they were calculated |

*Our web collection on statistics for biologists contains articles on many of the points above.*

## Software and code

Policy information about availability of computer code

| | |
|---|---|
| Data collection | Images were collected as 16-bit RGB stack files using a Zeiss LSM 880 confocal microscope equipped and the following lasers and filters were used: 488nm (491–553nm), 561nm (571–642) and 405nm (410-484). Except as noted, a single z-stack image from the cross-section with the largest oocyte diameter was acquired for each specimen. |
| Data analysis | Images were analyzed using Fuji (Image J 1.52n) software. a segmented circle was created in the middle of the zona pellucida, and the fluorescence intensity at each point on the line was determined. The data points were then imported into Excel (Build 12527.21416) and each point whose value was above the background value of the oocyte cytoplasm and higher than each of its immediately neighboring points was counted as a TZP. To achieve this, a formula was applied to each value, as indicated for the following example : =COUNTIFS(AD7, ">"&$AE $14,AD7, ">" &AD6,AD7, ">" &AD8). Here, AD6, AD7 and AD8 are three consecutive values (points on the line) and AE14 is the background value for the image - the formula counts AD7 as a TZP if its value is higher than both AD6 and AD8, as well as higher than AE14. AD8 is then compared to AD7 and AD9, as well as AE14, and so on.<br>To count the number of N-cadherin foci, images of the COCs were captured at a z-plane where the oocyte diameter was 50 µm, as the signal at the equator was weak owing to the presence of the cumulus cells. Using ImageJ, the oocyte cortex was traced using Freehand selections tool, and the number of foci of fluorescence above a threshold set at maximal intensity in oocyte cytoplasm were counted in this area. This value was divided by the size area to enable different specimens to be compared.<br>All statistical tests were performed using GraphPad Prism (9.0.0) |

For manuscripts utilizing custom algorithms or software that are central to the research but not yet described in published literature, software must be made available to editors and reviewers. We strongly encourage code deposition in a community repository (e.g. GitHub). See the Nature Research guidelines for submitting code & software for further information.

## Data

Policy information about availability of data

All manuscripts must include a data availability statement. This statement should provide the following information, where applicable:

- Accession codes, unique identifiers, or web links for publicly available datasets
- A list of figures that have associated raw data
- A description of any restrictions on data availability

All original data files will be made available upon request to the corresponding author.

# Field-specific reporting

Please select the one below that is the best fit for your research. If you are not sure, read the appropriate sections before making your selection.

- [x] Life sciences
- [ ] Behavioural & social sciences
- [ ] Ecological, evolutionary & environmental sciences

For a reference copy of the document with all sections, see nature.com/documents/nr-reporting-summary-flat.pdf

# Life sciences study design

All studies must disclose on these points even when the disclosure is negative.

| | |
|---|---|
| Sample size | No sample sizes were pre-determined. Cumulus-oocyte complexes (COCs) were collected from typically between 3 and 6 mice, and combined in one dish. They were then arbitrarily allocated to different treatment groups. Because the number of COCs obtained varied between experiments, not all replicates contained the same starting number of COCs. For each experimental treatment, at least three biological replicates were performed, meaning that the COCs were collected on different days and from different groups of mice. Each COC represents one independent sample. Therefore, this design permits a large n-value for each experimental condition. Because of the large n-value and the large differences in mean value observed, the p-values obtained are very low - in most cases less than 0.0001. |
| Data exclusions | No data were excluded from the study. |
| Replication | Each experimental treatment was evaluated using at least three biological replicates, meaning that the COCs were collected on different days and from different groups of mice. We endeavoured to have at least eight COCs per treatment group per experimental replicate. All replicates gave similar results and all were included in the data presented and analyzed. |
| Randomization | Cumulus-oocyte complexes (COCs) were collected from several mice and combined in one dish. They were then arbitrarily allocated to different treatment groups. Strictly speaking, this is not random; however, as differences between COCs are not evident at the low-power magnification used for collection and allocation, we believe that the allocation was effectively random. |
| Blinding | It was not possible to blind investigators to the identity of the different treatment groups. However, the role of the investigator was simply to record images using the confocal microscope, using pre-determined parameters. There was no subjective component to this task. All analysis was performed using the software and statistical programs described above. |

# Reporting for specific materials, systems and methods

We require information from authors about some types of materials, experimental systems and methods used in many studies. Here, indicate whether each material, system or method listed is relevant to your study. If you are not sure if a list item applies to your research, read the appropriate section before selecting a response.

## Materials & experimental systems

| n/a | Involved in the study | |
|---|---|---|
| [ ] | [x] | Antibodies |
| [x] | [ ] | Eukaryotic cell lines |
| [x] | [ ] | Palaeontology and archaeology |
| [ ] | [x] | Animals and other organisms |
| [x] | [ ] | Human research participants |
| [x] | [ ] | Clinical data |
| [x] | [ ] | Dual use research of concern |

## Methods

| n/a | Involved in the study | |
|---|---|---|
| [x] | [ ] | ChIP-seq |
| [x] | [ ] | Flow cytometry |
| [x] | [ ] | MRI-based neuroimaging |

## Antibodies

| | |
|---|---|
| Antibodies used | Beta-catenin - BD Transduction Laboratories 610153<br>E-cadherin - BD Transduction Laboratories 610181<br>N-cadherin - Abcam 18203 |

| | |
|---|---|
| | Red fluorescent protein - Cedarlane 600-401-379<br>TJP1 - Novus NBP1-85047<br>phosphorylated ERK - Cell Signaling Technologies 9106<br>TACC - Abcam 134154<br>Rabbit IgG-488 - ThermoFisher A11008<br>Mouse IgG-488 - ThermoFisher A11001 |
| Validation | Beta-catenin<br>- mfr website - IB: single band of expected size in cell lysate; IF: expected cortical localization<br>- our data - expected oocyte cortical localization, matches published studies (Mora, PMID 22321830)<br>E-cadherin<br>- mfr website - IB: single band of expected size in cell lysate; IF: expected cortical localization<br>- our data - expected oocyte cortical localization, matches published studies (Mora, PMID 22321830)<br>N-cadherin<br>- mfr website - IB: single band of expected size in cell lysate<br>- our data - expected TZP-oocyte interface localization, matches published studies (Mora, PMID 22321830)<br>Red fluorescent protein<br>- our data - stains cellular membranes of mTmG transgenic mice but not wild-type mice (El-Hayek, PMID 29576478)<br>TJP1<br>- mfr website - IB: single band of expected size in cell lysate; IF: IF: expected cellular localization<br>- our data - expected TZP-oocyte interface localization, matches published studies (Mora, PMID 22321830)<br>Phosphorylated ERK<br>- mfr website - IB: single band of expected size in cell lysate<br>- our data - single band of expected size in cell lysate - have used this antibody in many published papers<br>TACC<br>- mfr website - IB: single band of expected size in cell lysate<br>- our data - IB: single band of expected size in cell lysate |

## Animals and other organisms

Policy information about studies involving animals; ARRIVE guidelines recommended for reporting animal research

| | |
|---|---|
| Laboratory animals | CD-1 mice<br>-obtained from Charles River Canada<br>-females approximately 18-21 days old were used<br>-in most experiments, females received an injection of 5IU of eCG approximately 44 hr prior to collection of COCs. In some experiments, females received an inection of eCG followed 44 hr later by an injection of 5IU hCG, and COCs were recovered at 4 or 8 hr after hCG injection.<br><br>mTmG (membrane-Tomato/membrane-Green)<br>-founder mice were obtained (Jackson Laboratory, Bar Harbor, ME; strain 007676) and a colony established at the RI-MUHC<br>-females approximately 18-21 days olld were used.<br>-females received an injection of 5IU of eCG approximately 44 hr prior to collection of COCs. |
| Wild animals | Study did not involve wild animals. |
| Field-collected samples | Study did not involve samples collected from the field. |
| Ethics oversight | All procedures were approved by the Animal Care Committee of the Research Institute of the McGill University Health Centre under protocol 7783. |

Note that full information on the approval of the study protocol must also be provided in the manuscript.
